# Four Neotropical frog species exhibit shared and distinct skin bacterial communities in a laboratory setting

**DOI:** 10.17912/micropub.biology.002080

**Published:** 2026-03-09

**Authors:** Viraj Jansari, Daniela A. Castro-Martinez, Melody J. Dailey, Olivia Roti, Morgan R. Seibert, Baraa J. Abdelghne, Gwendolyne K. Aguilar, Anna Amine, Kaya Ben-Efraim, Riley E. Carolan, Ashley N. Carter, Melody Chang, Nicole J. Dye, Chantal A. Le, Massiel Melian, Keira C. Nakamura, Ruhee Nemawarkar, Amy T. Nguyen, Jessie Ong, Keshav Saigal, Harmony M. Sosa, Linh T. Vo, Samuel H. Wu, Zeinab K. Zreik, Griselda Morales, Halyna Kuznetsov, Dave Ramirez, Mila Pamplona-Barbosa, Madison P. Lacey, Nicole Bradon, Chloe L. Golde, Lauren A. O’Connell

**Affiliations:** 1 BIO161 Organismal Biology Lab, Stanford University, Stanford, California, United States; 2 Department of Biology, Stanford University, Stanford, California, United States

## Abstract

Amphibian skin microbiomes are an essential part of host physiology and pathogen defense. In this study, we identified common and distinct microbiota across four Neotropical frog species in laboratory conditions. Across frogs, we found communities dominated by *Pseudomonas* and *Chryseobacterium* . However, each frog species had a unique bacterial profile with at least one unique bacterial genus, highlighting the variability of naturally occurring amphibian skin microbiomes. These experiments were conducted by undergraduate students in an upper-division laboratory course, demonstrating how curiosity-based science education can lead to practical research experiences and new scientific insights.

**Figure 1. Common and distinct bacteria on the skin of Neotropical frogs in laboratory conditions:**
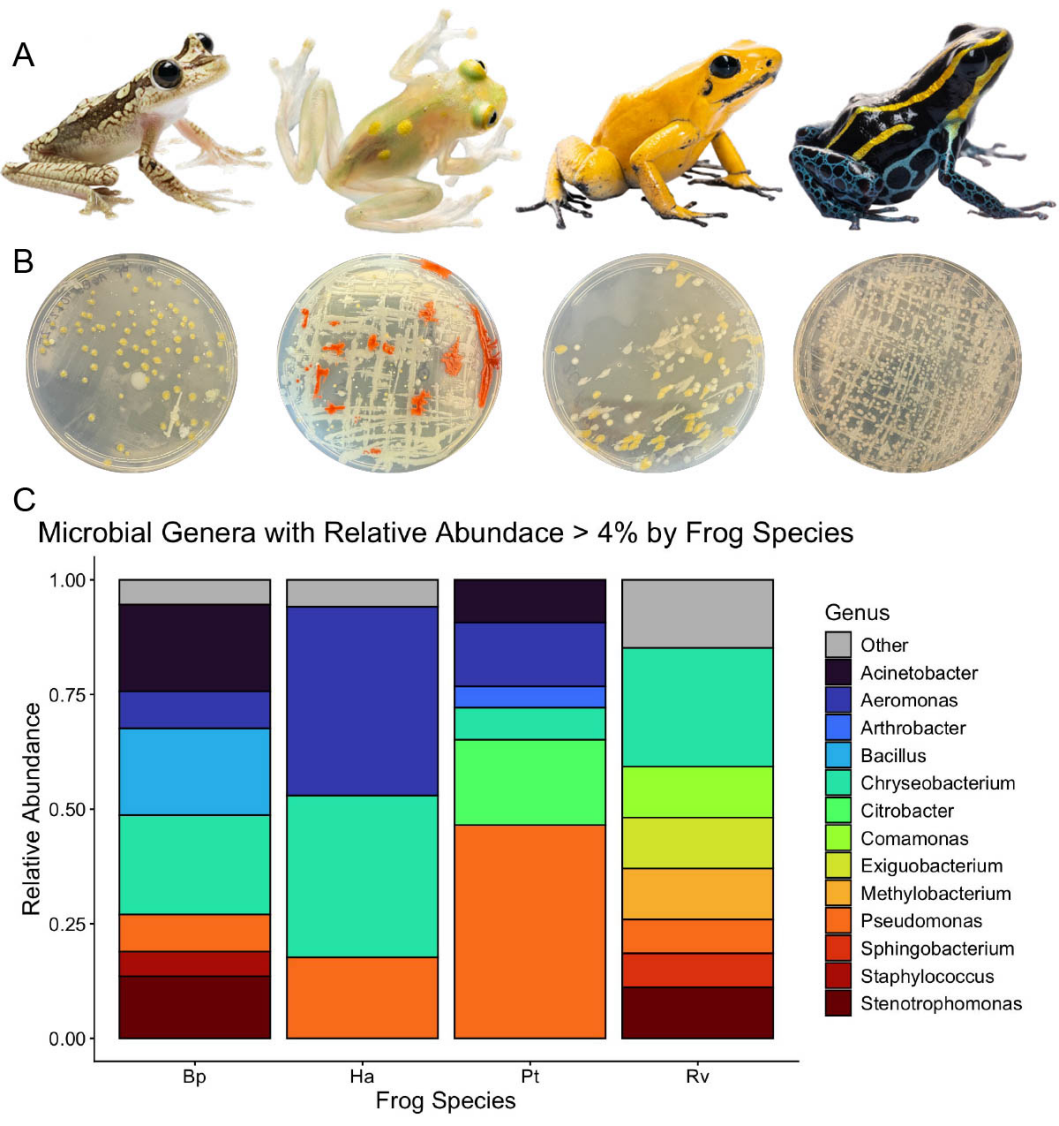
(A) The frogs used in this study, from left to right: the Imbabura tree frog ( *Boana picturata,* Bp), the sun glass frog ( *Hyalinobatrachium aureoguttatum,* Ha), the golden poison frog ( *Phyllobates terribilis,* Pt), and the variable poison frog ( *Ranitomeya variabilis,* Rv). (B) Examples of tryptone plates streaked with skin swabs taken from each frog species, in the same order as (A). (C) Relative abundance plot showing the bacterial genera of each frog species. Bacteria with a relative abundance of less than 4% are grouped in the “other” category (gray).

## Description

The microbiomes associated with organisms play substantial roles in both health outcomes and disease susceptibility (Peixoto et al., 2021). In amphibians, the skin microbiome is critical for host physiology, serving functions as diverse as the production of odorants involved in social activity to immunity against fungal pathogens such as chytrid ( *Batrachochytrium dendrobatidis)* , which poses a major threat to amphibian species worldwide (Harris et al., 2009; Ross et al., 2019). Despite its essential role in host health, the amphibian skin microbiome remains relatively understudied across diverse host species. Here, we characterize the composition of the bacterial skin microbiome of four Neotropical frog species ( Figure 1A ): the Imbabura tree frog ( *Boana picturata* ), the sun glass frog ( *Hyalinobatrachium aureoguttatum)* , the golden poison frog ( *Phyllobates terribilis)* , and the variable poison frog ( *Ranitomeya variabilis)* . We tested the hypothesis that there would be some common bacteria across all four frog species due to similar laboratory environments, but innate species differences would select for some distinct bacteria.

To characterize the skin microbiome of each frog species, we swabbed six frogs of each species and streaked the microbes on tryptone plates ( Figure 1B ). After multiple rounds of colony streaking, we used PCR to sequence the 16S gene, which is commonly used to identify bacteria to the family or genus level (Johnson et al., 2019). The frog species varied in the diversity of the microbes detected with this method, where the sun glass frog ( *H. aureoguttatum* ) had the fewest unique genera ( Figure 1B ). The greatest number of unique bacterial genera was found in the variable poison frog ( *R. variabilis* ), who also had the highest number of genera with only one appearance (the “other” category). Four bacterial genera with relative abundance > 4% were detected only in *R. variabilis* , including *Comamonas* , *Exigiobacterium* , *Methylobacterium* , and *Sphingobacterium* , which have all been detected in poison frogs sampled in nature (Caty et al., 2025; Fischer et al., 2025). All other bacteria with relative abundance above 4% were found in multiple species with the exception of *Bacillus* detected in the Imbabura tree frog ( *B. picturata* ) *,* and *Arthrobacter* and *Citrobacter* detected in the golden poison frog *&nbsp;* ( *P. terribilis* ). Overall, this suggests species differences may influence the variability of their skin microbial diversity. For example, *B. picturata* produces skin peptides with antimicrobial activity that likely impacts their bacterial community (Morán-Marcillo et al., 2022). Overall, these results are in line with other work in amphibians that indicates host phylogeny, in addition to environment, influences the skin microbiome composition (Ramírez-Barahona et al., 2023; Caty et al., 2025).

Two bacterial genera with relative abundance > 4% were detected in all four frog species: *Pseudomonas* (Gammaproteobacteria) and *Chryseobacterium* (Flavobacteriia). Both genera have been reported in frog microbiomes, with *Pseudomonas* being one of the most frequently detected (Bresciano et al., 2015; Rebollar et al. 2019), including in skin microbiomes of poison frogs (Caty et al., 2025; Fischer et al., 2025). The shared *Chryseobacterium* bacteria has also been detected in other amphibians, including newts (Kirk et al., 2013), clawed frogs ( *Xenopus* ; Chapman et al., 2024) and poison frogs (Caty et al., 2025). In *Xenopus* tadpoles, the presence of *Chryseobacterium* is associated with improved regeneration (Chapman et al., 2022), suggesting a role in improved health outcomes. Moreover, some species of *Pseudomonas* and *Chryseobacterium* have been found to inhibit fungal pathogens, including *Batrachochytrium dendrobatidis* (Woodhams et al., 2016; Martin et al., 2019). The shared bacterial genera suggest that, regardless of phylogenetic differences, amphibians share a fundamental layer of overlap in microbial communities that may play a role in regulating health and disease.

Although all frogs were housed in the same animal facility, the terraria environment was specific to each frog species, where poison frogs have an environment mimicking the leaf litter forest floor while tree frogs were provided with more arboral structures and additional water features necessary for breeding. Therefore, we predicted that terrestrial poison frogs would be more similar to each other than the arboreal tree frogs due to relatedness and differences in lifestyle/terraria. The frogs with the greatest microbial overlap was the glass frog *H. aureoguttatum* compared to the poison frog *P. terribilis* and the tree frog *B. picturata* , although this is likely due to the glass frog being the lowest variation in skin bacterial genera. Indeed, these three frog species shared *Aeromonas* , which is commonly present in water. This matches the environments of the frogs in the laboratory, as the three frog species with *Aeromonas* have access to large water pools in their terraria whereas *R. variabilis* does not (only small water cups are provided for tadpoles). Work in Malagasy and Neotropical frogs found that the host environment was a stronger determinant of skin microbiome than phylogeny (Bletz et al., 2017; Varela et al., 2018; Caty et al., 2025). Other amphibian studies report that host species is the most influential factor in skin microbial community assemblages, but habitat and life stage also play a role in determining microbiome composition (Kueneman et al., 2014). We cannot disentangle the effects of host environment and phylogeny on skin microbiome variability in this study given the terraria components also differed across species. Further investigation is needed to untangle the impact of environment and host genetics on skin microbiome communities in amphibians.&nbsp;

In summary, the bacterial makeup of the skin microbiome of the frog species tested here is consistent with other research characterizing the composition of the amphibian skin microbiome. For future studies of captive frog species, collection of environmental samples in frog terraria would clarify how different laboratory terraria designs contribute to species differences in skin microbiomes. Finally, we note that the microbial diversity of frogs in laboratory conditions is likely much lower than frogs in nature (Caty et al., 2025), which should be quantified in future studies to better understand the skin microbial diversity in nature and how this diversity impacts health and disease in wild amphibian populations.

## Methods

*Animals*

The frogs used in this study were adults and maintained for research purposes in the O’Connell Lab at Stanford University. Animals were housed in non-sterile glass terraria with other frogs of the same species. The following Neotropical frog species were used in this study: the golden poison frog ( *Phyllobates terribilis)* , the variable poison frog ( *Ranitomeya variabilis)* , the sun glass frog ( *Hyalinobatrachium aureoguttatum)* , and the Imbabura tree frog ( *Boana picturata)* . Six adult individuals from separate tanks of each species were used in this study. Terraria for poison frogs contained sphagnum moss, live philodendrons, dried leaves, shelters (loose leaves for *R. variabilis* and cocohuts for *P. terribilis* ), and water pools (large water pool dishes for *P. terribilis* and small water pools in film canisters for *R. variabilis* ). For the Imbabura tree frogs, terraria contained live plants (philodendrons, monsteras, and dracaenas), manzanita wood for climbing structures, and large water pools. Glass frogs were housed in paludariums with running water, manzanita wood, sphagnum moss, and live plants (philodendrons and monsteras). Frogs were kept on a 12:12 h light cycle and fed wingless *Drosophila* fruit flies or crickets dusted with vitamin supplements three times per week. All procedures were approved by the Institutional Animal Care and Use Committee at Stanford University (protocol # 34959 ).

*Skin microbe collection and growth*

Frog skin microbe samples were collected as detailed in Caty et al. (2024 & 2025). Briefly, skin microbe samples were collected using sterile cotton swabs. Each individual was rinsed with sterile water to remove transient microbes and then swabbed with 10 strokes on the dorsal and ventral surfaces. The swabs were then placed on ice until they could be struck onto 1% tryptone agar plates. Plates were sealed with parafilm and incubated for one week at 25 °C. Plates were imaged and colony morphology was described by students. Then, 14 colonies were picked from each plate to restreak and isolate. This isolation procedure was repeated once 3 days later in order to have a single microbial colony for microbial DNA isolation.&nbsp;

*16S PCR and Sanger sequencing*

Microbial DNA was isolated using Extraction Solution (Millipore Sigma E7526). Cell lysis was performed in 20 µL of RedExtract Extraction Buffer for 6 minutes at 65°C, followed by 3 minutes at 95 °C. After lysis, 20 µL of Neutralization Buffer B (Millipore Sigma N3910) was added, and samples were placed on ice. Then, 4 µL of extracted DNA was added to 16 µL REDExtract-N-Amp PCR ReadyMix (Millipore Sigma R4775). The 16S rRNA gene was amplified using the 27F (5’-AGA GTT TGA TCM TGG CTC AG-3’) and 1492R (5’-TAC GGY TAC CTT GTT AYG ACT T-3’) primers synthesized by Elim Biopharmaceuticals (Hayward, CA, USA). The 16S rRNA PCR parameters were as follows: denaturation at 94 °C for 30 s, followed by 30 cycles of denaturation at 94 °C for 15 s, annealing at 55 °C for 30 s, and extension at 65 °C for 2 min with a final extension of 5 min at 65 °C. PCR products were cleaned up using HT ExoSAP-IT Fast High-Throughput PCR Product Cleanup kit (ThermoFisher Scientific) according to manufacturer’s directions. Samples were sent to Elim Biopharmaceuticals for Sanger sequencing. Forward and reverse reads were aligned using Geneious Prime using the following parameters: sequences were trimmed to an error probability limit of 0.05, then alignment was made using global alignment with free end gaps, cost matrix of 65% similarity (5.0/-4.0), gap open penalty 12 and gap extension penalty 3, with automatically determine direction selected. Sequences were BLASTed using the Megablast tool in Geneious Prime to the NCBI 16S ribosomal RNA database.&nbsp;

*Classroom pedagogy&nbsp;*

The experiments in this study were performed over five laboratory sessions. These sessions began with students swabbing frogs for microbes under staff and veterinarian supervision, and then streaking swabs on plates. The next laboratory session involved characterizing microbial growth on plates (classifying morphotypes) and picking colonies to restreak for DNA isolation. This activity was preceded by a safety discussion on zoonotic potential of microbes and safety precautions needed, including the use of masks and working in a laminar flow hood. The next two laboratory sessions were used for PCR and gel electrophoresis. The final laboratory session was used to analyze sequencing results and compile data. Weekly homework included reading relevant literature, analysis, and visualization of data collected by the entire class, and writing individual drafts of a journal-style article, which were combined into this article by the instructors. Assignments were graded as complete/incomplete. Students received detailed feedback on their data visualization and writing at each stage. All students edited and approved of the final manuscript.

## Data Availability

Description: Excel File with 16S sequencing data for each bacteria isolate. Resource Type: Dataset. DOI: https://doi.org/10.22002/9frk4-cez09
